# Birmingham mid-head resection periprosthetic fractures: Case report

**DOI:** 10.1016/j.ijscr.2019.10.012

**Published:** 2019-10-12

**Authors:** Inés Fraile Gamarra, Juan Fernando Jiménez Viseu Pinheiro, Carlos Cano Gala, Juan Francisco Blanco Blanco

**Affiliations:** Complejo Asistencial Universitario de Salamanca, Paseo de San Vicente 58-182, 37001, Spain

**Keywords:** Case report, Birmingham, Periprosthetic fracture, Subtrochanteric, Mid-Head resection, Total hip arthroplasty

## Abstract

**Introduction:**

Total hip arthroplasty in young patients can cause problems when it comes to choosing a suitable implant. The Birmingham Mid-Head Resection prosthesis (BMHR) offers the option of preserving bone stock despite its poor quality in the femoral neck. Femoral neck fractures are a known complication of hip resurfacing prostheses and the main reason for revision surgery. Retaining the femoral implant may cause difficulties in osteosynthesis carried out with implants habitually used for intertrochanteric or femoral neck fractures (e.g., a screw-plate device or a cephalomedullary nail) [2].

**Presentation of case:**

We present the case of an 81-year-old patient who underwent surgery for right hip osteoarthritis and received a Birmingham Mid-Head Resection prosthesis. She had no history of previous fractures due to frailty. While on holiday, she sustained a periprosthetic fracture as a result of a fall. While the most common fracture in resurfacing prosthesis is produced in the femoral neck, in this case the patient sustained a subtrochanteric fracture. This fracture was surgically treated by means of open reduction and internal fixation with trochanteric plate and three cerclages.

**Discussion:**

Olsen et al. [8] described two fracture patterns: transcervical vertical shear type and subcapital type. In our patient's case, the fracture pattern was different to those described, as the fracture started in the cervical area and reached the subtrochanteric area. This change in the standard periprosthetic fracture pattern leads to a change in the therapeutic attitude that must be adopted.

**Conclusion:**

BMHR prostheses are metal-on-metal implants that resulted from the development of the standard resurfacing prostheses used to treat hip osteoarthritis in young patients with the goal of preserving as much bone stock as possible.

In this paper we will describe a rare complication in this type of prosthesis and how it was surgically treated after reviewing the available literature.

## Highlights

• Birmingham Mid-Head Resection (BMHR) prostheses are implants used to treat hip osteoarthritis in young patients with the goal of preserving as much bone stock as possible.
• Compared to total hip arthroplasty, BMHR prostheses offer advantages such as lower rate of dislocation and lower rate of infection and sepsis.
• The revision rate of BMHR devices is higher than that of conventional total hip arthroplasty devices. Furthermore, these revisions occur earlier with the former.
• The main complication of BMHR prostheses are femoral neck fratures, which are also the most common reason for revision.
• Patterns of BMHR periprosthetic fractures usually described in the literature are subcapital and transcervical ones. Nevertheless, more patterns can be found and different therapeutic attitudes must be adopted.

## 1 Introduction

This work has been reported in line with the SCARE criteria [1].

Hip arthroplasty in young adults remains a challenge for orthopaedic surgeons. The need for implants that last longer and are better suited to an increasingly active lifestyle poses difficulties when choosing a suitable prosthesis. The knowledge that surgery at a young age increases the probability of future revision has led to the development of bone-preserving resurfacing arthroplasty as opposed to conventional total hip replacement. However, resurfacing relies on bone quality. Poor bone quality is a relative contraindication to resurfacing arthroplasty. Therefore, we must be particularly selective when choosing patients for this type of surgery. Patients who were initially considered for resurfacing surgery but were excluded due to poor bone quality in the femoral neck now have the option of using a Birmingham Mid-Head Resection (BMHR) prosthesis, which is a metal-on-metal implant with an uncemented short stem. BMHR prostheses are implanted after removing the deficient femoral head and stabilising its base; this is different from conventional resurfacing, in which a metal resurfacing 'cap' is placed over the femoral head.

This implant is a novel solution for this group of patients. There is currently very little literature, with survival results published at a follow-up of 1.2–5.3 years [3,4].

Femoral neck fractures are the most frequent complication of this type of prosthesis, as well as the main reason for revision. According to a study carried out by Brennan et al., the femoral neck fracture rate in the first year following hip resurfacing has been reported as 1.31%. Factors that contribute to the risk of fracture are female gender, a high BMI and osteoporosis. Surgical factors such as femoral neck notching and a malpositioned femoral component may also contribute [5].

Other common complications of this type of prosthesis are pain, infection, femoral head collapse, effusion and acetabular osteolysis [6].

According to a systematic review conducted by Marshall et al. [7], the revision rate of metal-on-metal hip resurfacing devices is higher than that of conventional total hip arthroplasty devices. Furthermore, these revisions occur earlier with the former. However, dislocation rates are higher in the latter. Infections/sepsis are more frequent in total hip arthroplasty than in metal-on-metal hip resurfacing, as is the case with femoral neck fractures.

## 2 Case report

**Fig. 1:**
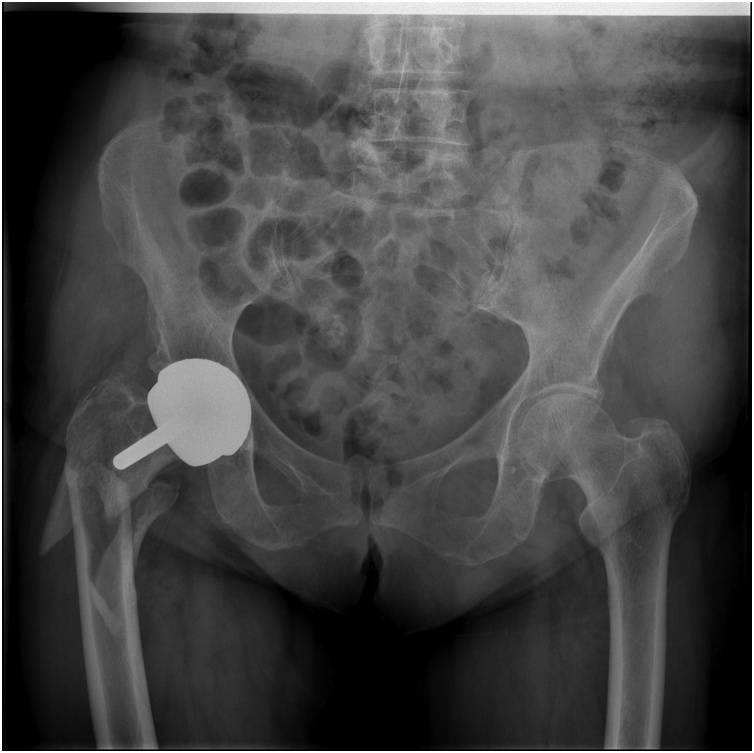
Periprosthetic fracture.

We present the case of an 81-year-old patient from England with a history of arterial hypertension, atrial fibrillation anticoagulated with Warfarin, and obesity. She also took antihypertensive drugs and beta-blockers, but no bisphosphonates. She had no history of previous fractures due to fragility. She had previously undergone right total hip resurfacing arthroplasty due to osteoarthritis when she was 65. While on holiday, she fell down some stairs and was sent to A&E due to pain and functional deficit of the right hip. During the examination, it was discovered that her right lower limb was shortened and externally rotated. There were no distal neurovascular alterations. Plain radiographs revealed a periprosthetic fracture with a spiral pattern, which started in the cervical area and reached the subtrochanteric area (Fig. 1).

**Fig. 2:**
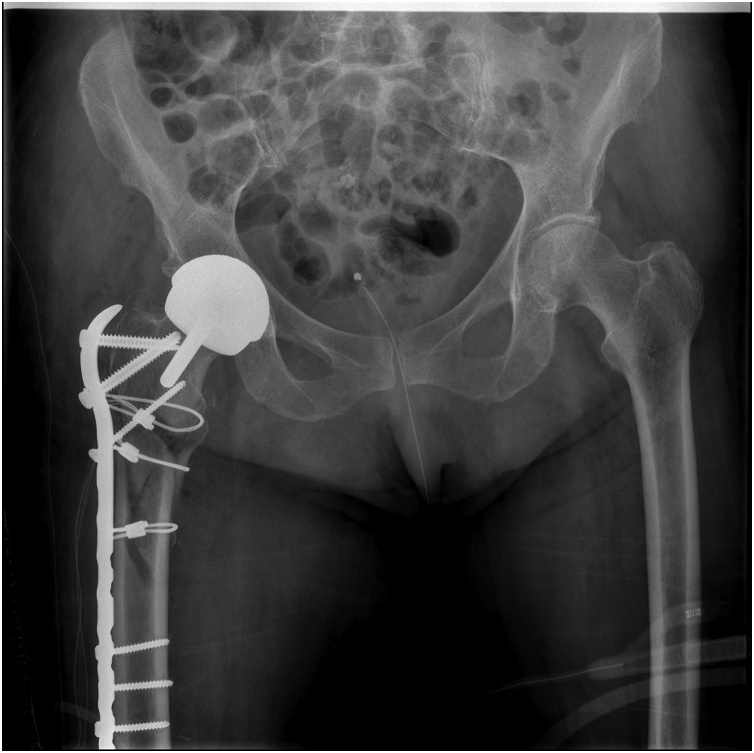
Postoperative radiograph. Internal fixation with trochanteric plate and cerclages.

**Fig. 3:**
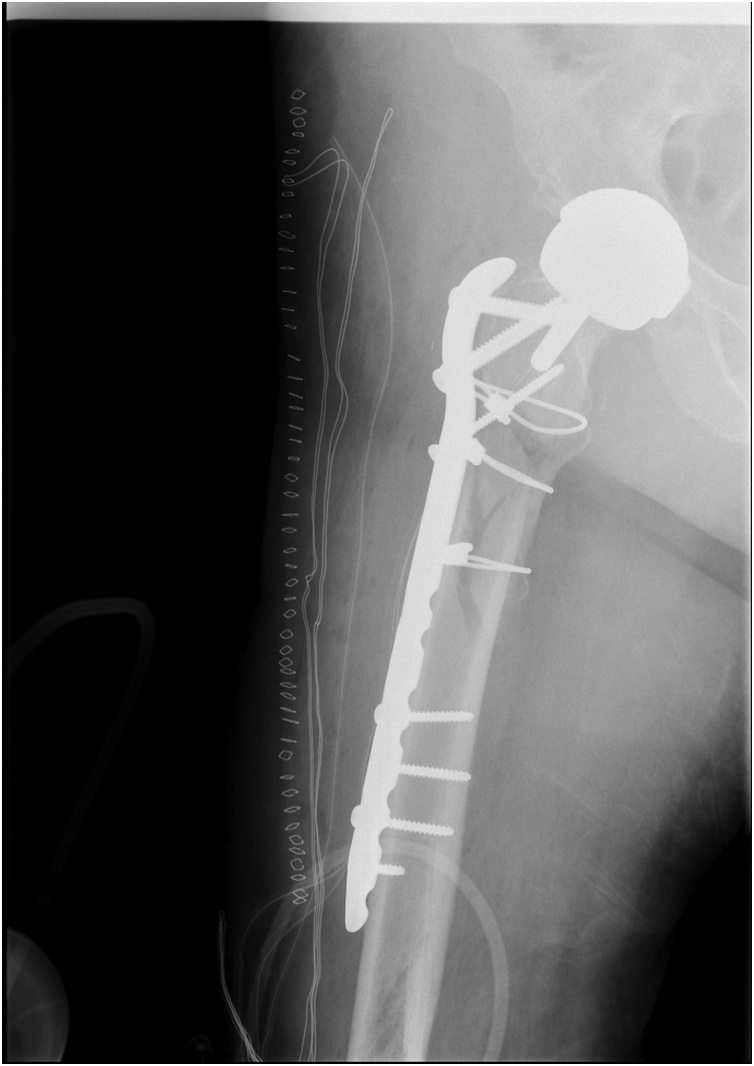
Postoperative radiograph. Internal fixation with trochanteric plate and cerclages.

After the 4-day Warfarin washout period, the patient's fracture was surgically treated by means of open reduction and internal fixation with trochanteric plate and three cerclages. The prosthesis was not removed, as it was not mobilized. Postoperative radiographs revealed a satisfactory reduction of the fracture (Fig. 2, Fig. 3).

Postoperative recommendation given to patient was walking with crutches in discharge of the limb affected for two months, and after that progressive support.

The follow up period of this patient was limited because of her nationality (she returned to England one month after surgery). On his first consultation, three weeks postoperative, wound was well healing with no evidence of infection and radiographic control was satisfactory, but the patient should continue in discharge of that limb for five weeks more.

## 3 Discussion

We present a case of periprosthetic fracture of a patient with a BMHR prosthesis. Olsen et al. [8] described two fracture patterns: transcervical vertical shear type and subcapital type. In our patient's case, the fracture pattern was different to those described, as the fracture started in the cervical area and reached the subtrochanteric area.

Cadaveric studies show that periprosthetic fracture rates are reduced when standard resurfacing prostheses are placed at a slight valgus angle, rather than in anatomical position. However, an excessive valgus angle may result in notching of the femoral neck, and cause a periprosthetic fracture too [9]. Given that the BMHR prosthesis is biomechanically similar to standard resurfacing implants, we can assume that a slight valgus placement of the BMHR protects against fractures, though there is no strong evidence in the literature. Recent studies show that a valgus placement does not strengthen the femur, and a varus placement does not weaken it [9,12]; however, a valgus placement does seem to have a protective effect against fractures.

It has been proven that a notch of over 2 mm in the femoral neck contributes to the fracture [4,10,11].

The BMHR prosthesis is an uncemented, metaphyseal fixed implant, which is different from the conventional epiphyseal implant. The shape of the BMHR femoral stem is conical and allows for physiologic loading similar to that of the intact femur [13,14].

## 4 Conclusions

BMHR prostheses are metal-on-metal implants that resulted from the development of the standard resurfacing prostheses used to treat hip osteoarthritis in young patients with the goal of preserving as much bone stock as possible. Compared to total hip arthroplasty, they offer advantages such as a lower rate of dislocation, as well as a lower rate of infection and sepsis. The main complications of this type of implant are femoral neck fractures, which are also the most common reason for revision.

The importance of this case stems from the fact that the periprosthetic fracture pattern differs from those usually described in the literature, as it is neither subcapital nor transcervical, but reaches the subtrochanteric area. This change in the standard periprosthetic fracture pattern leads to a change in the therapeutic attitude that must be adopted. From the literature limitation with no registry for subtrochanteric fracture surrounding BMHR implants, we show our manage and results that need further research to achieve solid knowledge about the approach and management for this kind of periprosthetic fracture.

## Declaration of Competing Interest

The authors declare that there is no conflict of interest.

## Sources of funding

The authors received no financial support for the research, authorship, and/or publication of this article.

## Ethical approval

Our study is exempt from ethical approval.

## Consent

Written informed consent was obtained from the patient for publication of this case report and accompanying images. A copy of the written consent is available for review by the Editor-in-Chief of this journal on request

## Author contribution

- Inés Fraile Gamarra: manuscript redaction, data collection, literature review.
- Juan Fernando Jiménez Viseu Pinheiro: study concept, manuscript redaction, literature review.
- Carlos Cano Gala: manuscript revision.
- Juan Francisco Blanco Blanco: manuscript revision.

## Registration of research studies

This is not a human study nor a clinical trial.

## Guarantor

Inés Fraile Gamarra.

Juan Fernando Jiménez Viseu Pinheiro.

## Provenance and peer review

Not commissioned, externally peer-reviewed.
